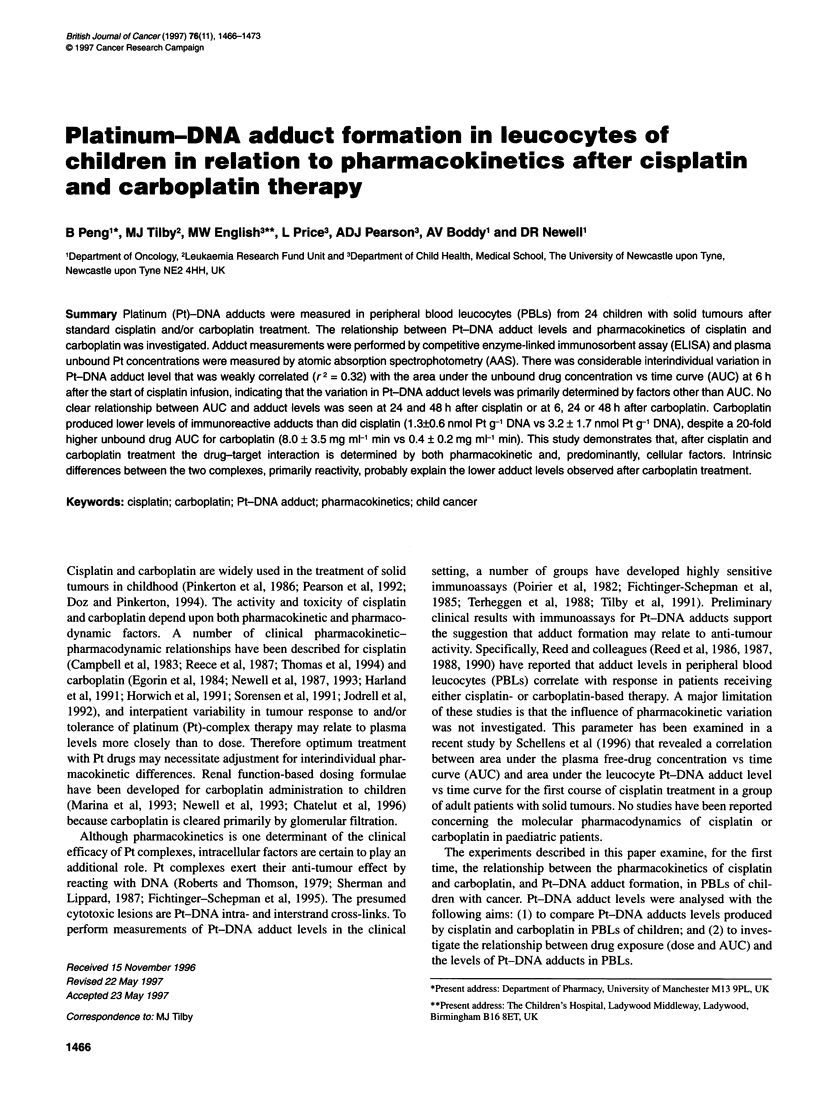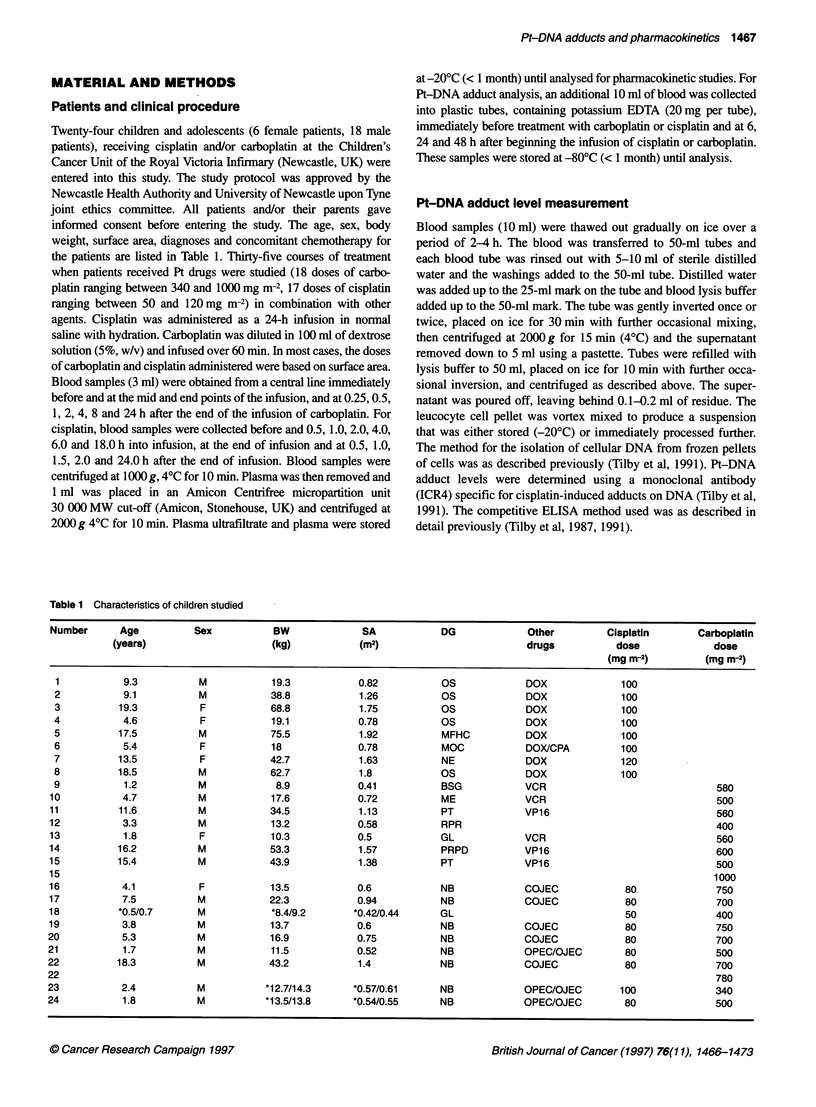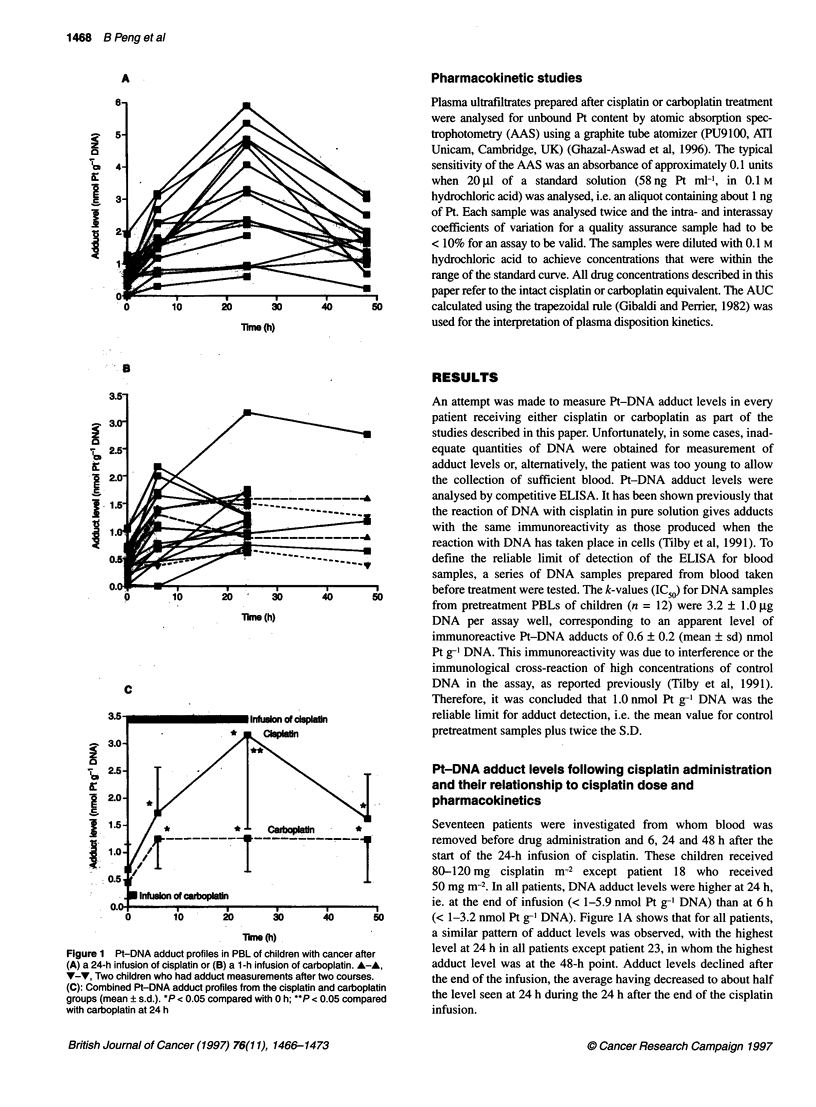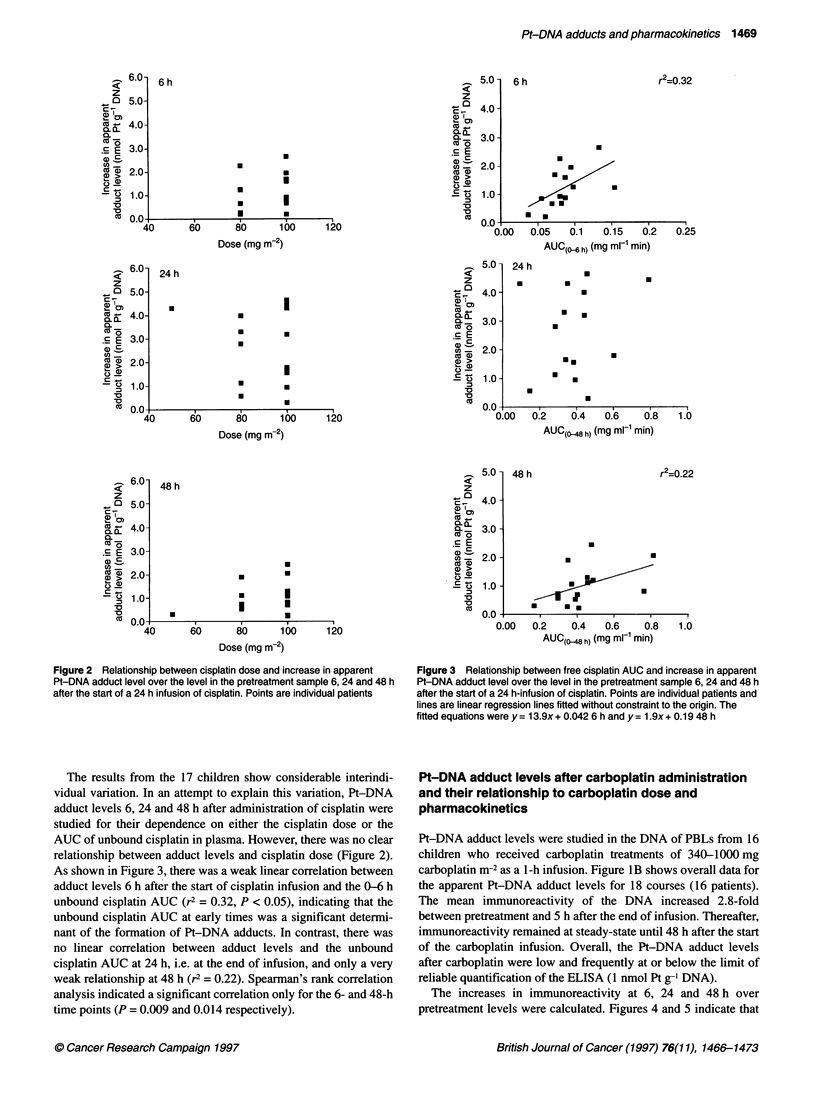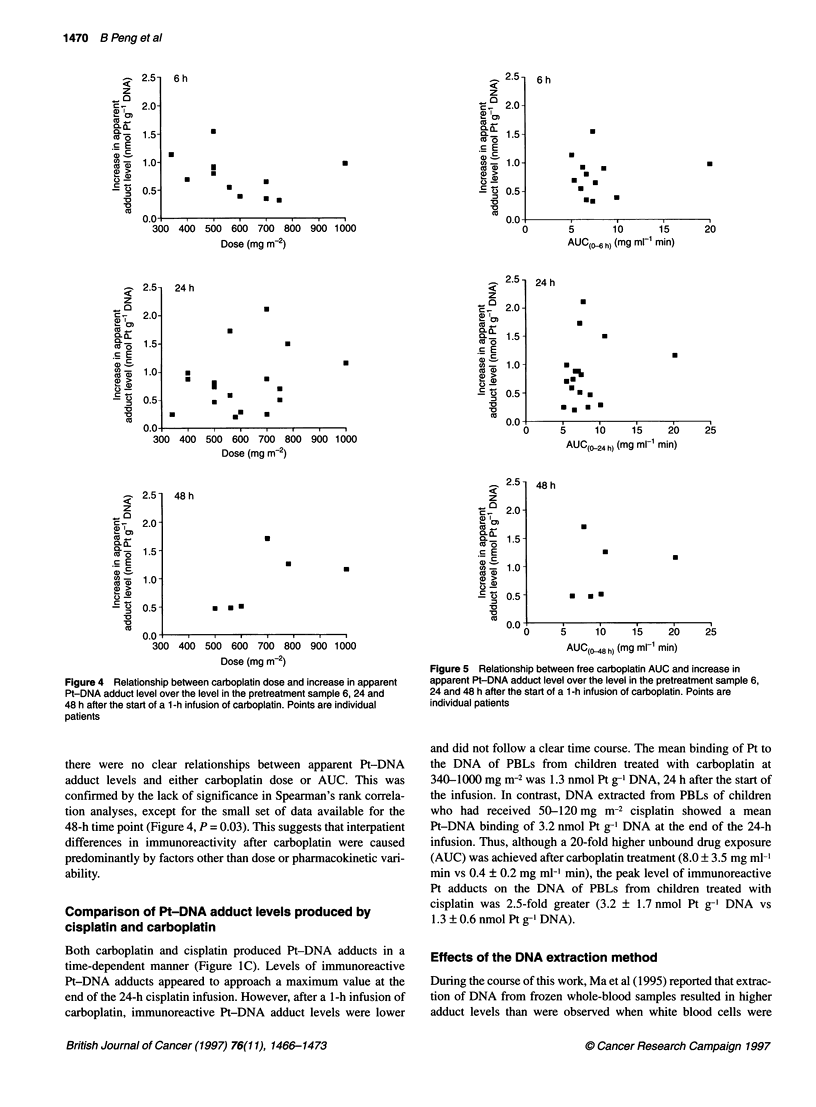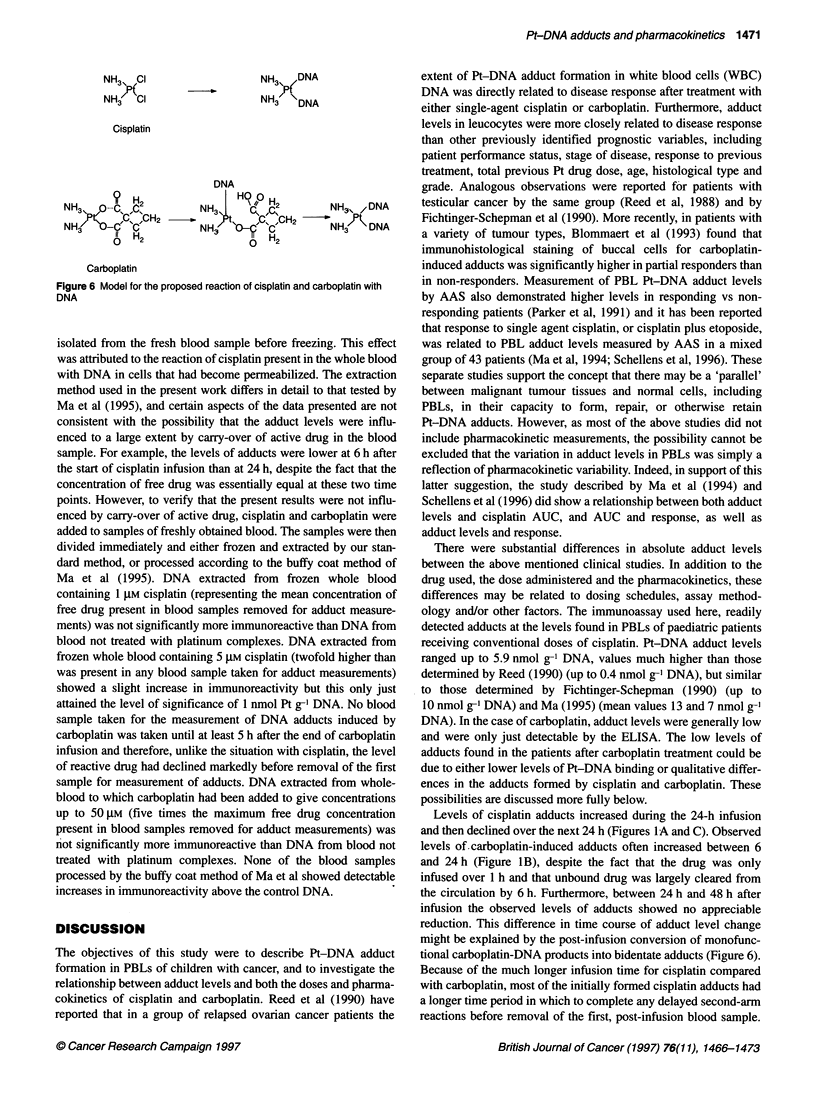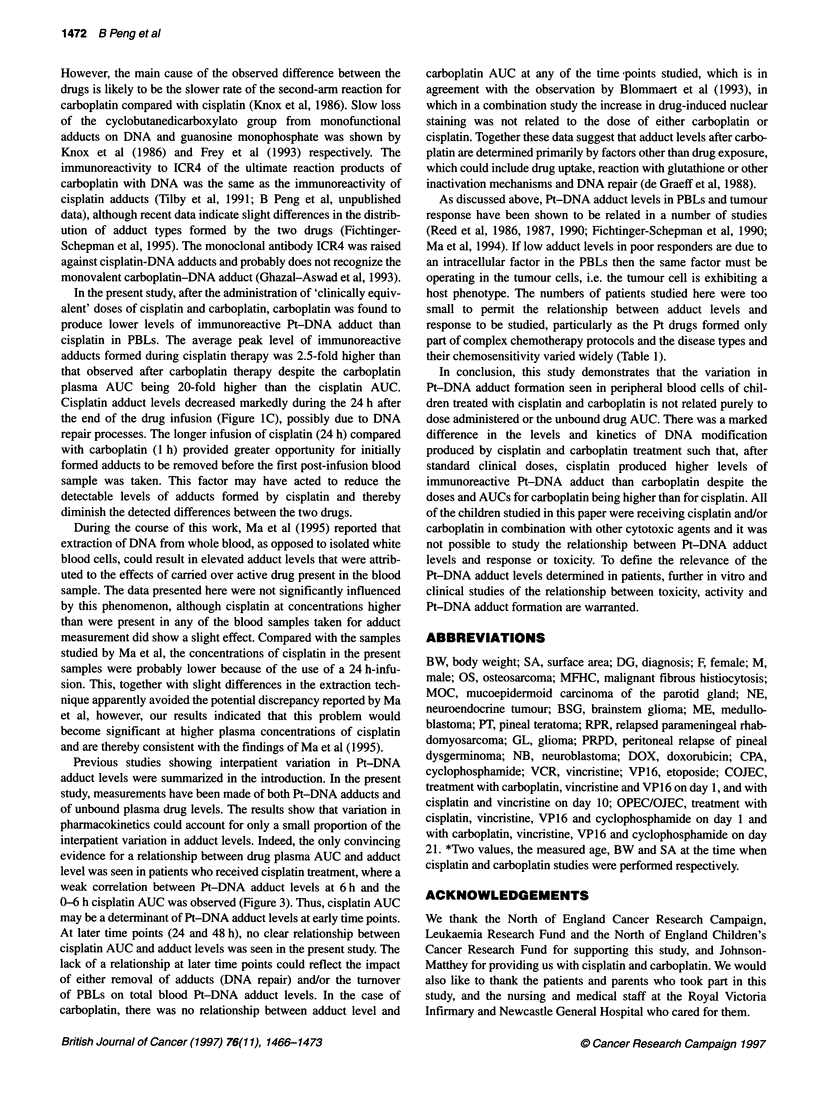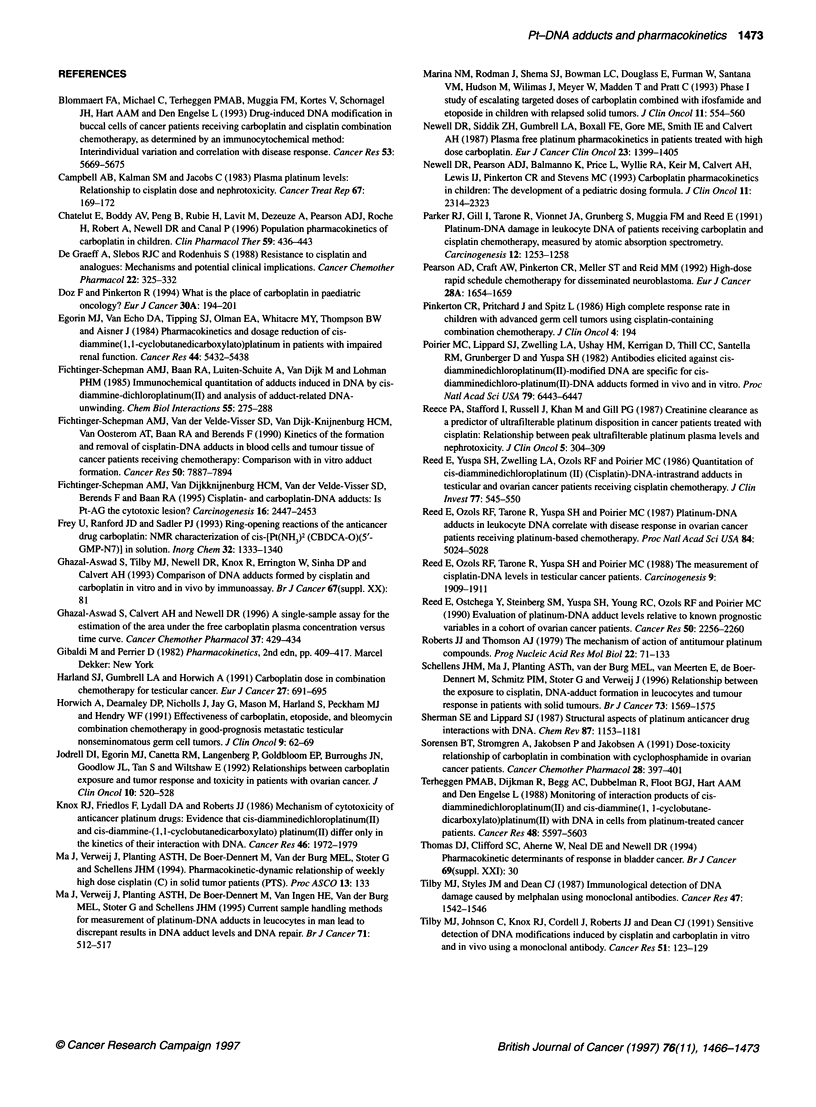# Platinum-DNA adduct formation in leucocytes of children in relation to pharmacokinetics after cisplatin and carboplatin therapy.

**DOI:** 10.1038/bjc.1997.579

**Published:** 1997

**Authors:** B. Peng, M. J. Tilby, M. W. English, L. Price, A. D. Pearson, A. V. Boddy, D. R. Newell

**Affiliations:** Department of Oncology, Medical School, The University of Newcastle upon Tyne, UK.

## Abstract

**Images:**


British Journal of Cancer (1997) 76(11), 1466–1473
© 1997 Cancer Research Campaign

### Platinum–DNA adduct formation in leucocytes of children in relation to pharmacokinetics after cisplatin and carboplatin therapy

**B Peng[1]*, MJ Tilby[2], MW English[3]**, L Price[3], ADJ Pearson[3], AV Boddy[1] and DR Newell[1]**

[1]Department of Oncology, [2]Leukaemia Research Fund Unit and [3]Department of Child Health, Medical School, The University of Newcastle upon Tyne, Newcastle upon Tyne NE2 4HH, UK

**Summary** Platinum (Pt)–DNA adducts were measured in peripheral blood leucocytes (PBLs) from 24 children with solid tumours after standard cisplatin and/or carboplatin treatment. The relationship between Pt–DNA adduct levels and pharmacokinetics of cisplatin and carboplatin was investigated. Adduct measurements were performed by competitive enzyme-linked immunosorbent assay (ELISA) and plasma unbound Pt concentrations were measured by atomic absorption spectrophotometry (AAS). There was considerable interindividual variation in Pt–DNA adduct level that was weakly correlated ($r^2 = 0.32$) with the area under the unbound drug concentration vs time curve (AUC) at 6 h after the start of cisplatin infusion, indicating that the variation in Pt–DNA adduct levels was primarily determined by factors other than AUC. No clear relationship between AUC and adduct levels was seen at 24 and 48 h after cisplatin or at 6, 24 or 48 h after carboplatin. Carboplatin produced lower levels of immunoreactive adducts than did cisplatin (1.3±0.6 nmol Pt $g^{-1}$ DNA vs 3.2 ± 1.7 nmol Pt $g^{-1}$ DNA), despite a 20-fold higher unbound drug AUC for carboplatin (8.0 ± 3.5 mg $ml^{-1}$ min vs 0.4 ± 0.2 mg $ml^{-1}$ min). This study demonstrates that, after cisplatin and carboplatin treatment the drug–target interaction is determined by both pharmacokinetic and, predominantly, cellular factors. Intrinsic differences between the two complexes, primarily reactivity, probably explain the lower adduct levels observed after carboplatin treatment.

**Keywords:** cisplatin; carboplatin; Pt–DNA adduct; pharmacokinetics; child cancer

Cisplatin and carboplatin are widely used in the treatment of solid tumours in childhood (Pinkerton et al, 1986; Pearson et al, 1992; Doz and Pinkerton, 1994). The activity and toxicity of cisplatin and carboplatin depend upon both pharmacokinetic and pharmacodynamic factors. A number of clinical pharmacokinetic–pharmacodynamic relationships have been described for cisplatin (Campbell et al, 1983; Reece et al, 1987; Thomas et al, 1994) and carboplatin (Egorin et al, 1984; Newell et al, 1987, 1993; Harland et al, 1991; Horwich et al, 1991; Sorensen et al, 1991; Jodrell et al, 1992), and interpatient variability in tumour response to and/or tolerance of platinum (Pt)-complex therapy may relate to plasma levels more closely than to dose. Therefore optimum treatment with Pt drugs may necessitate adjustment for interindividual pharmacokinetic differences. Renal function-based dosing formulae have been developed for carboplatin administration to children (Marina et al, 1993; Newell et al, 1993; Chatelut et al, 1996) because carboplatin is cleared primarily by glomerular filtration.

Although pharmacokinetics is one determinant of the clinical efficacy of Pt complexes, intracellular factors are certain to play an additional role. Pt complexes exert their anti-tumour effect by reacting with DNA (Roberts and Thomson, 1979; Sherman and Lippard, 1987; Fichtinger–Schepman et al, 1995). The presumed cytotoxic lesions are Pt–DNA intra- and interstrand cross-links. To perform measurements of Pt–DNA adduct levels in the clinical setting, a number of groups have developed highly sensitive immunoassays (Poirier et al, 1982; Fichtinger-Schepman et al, 1985; Terheggen et al, 1988; Tilby et al, 1991). Preliminary clinical results with immunoassays for Pt–DNA adducts support the suggestion that adduct formation may relate to anti-tumour activity. Specifically, Reed and colleagues (Reed et al, 1986, 1987, 1988, 1990) have reported that adduct levels in peripheral blood leucocytes (PBLs) correlate with response in patients receiving either cisplatin- or carboplatin-based therapy. A major limitation of these studies is that the influence of pharmacokinetic variation was not investigated. This parameter has been examined in a recent study by Schellens et al (1996) that revealed a correlation between area under the plasma free-drug concentration vs time curve (AUC) and area under the leucocyte Pt–DNA adduct level vs time curve for the first course of cisplatin treatment in a group of adult patients with solid tumours. No studies have been reported concerning the molecular pharmacodynamics of cisplatin or carboplatin in paediatric patients.

The experiments described in this paper examine, for the first time, the relationship between the pharmacokinetics of cisplatin and carboplatin, and Pt–DNA adduct formation, in PBLs of children with cancer. Pt–DNA adduct levels were analysed with the following aims: (1) to compare Pt–DNA adducts levels produced by cisplatin and carboplatin in PBLs of children; and (2) to investigate the relationship between drug exposure (dose and AUC) and the levels of Pt–DNA adducts in PBLs.

*Received 15 November 1996*
*Revised 22 May 1997*
*Accepted 23 May 1997*

*Correspondence to:* MJ Tilby

*Present address: Department of Pharmacy, University of Manchester M13 9PL, UK

**Present address: The Children's Hospital, Ladywood Middleway, Ladywood, Birmingham B16 8ET, UK

### MATERIAL AND METHODS

### Patients and clinical procedure

Twenty-four children and adolescents (6 female patients, 18 male patients), receiving cisplatin and/or carboplatin at the Children's Cancer Unit of the Royal Victoria Infirmary (Newcastle, UK) were entered into this study. The study protocol was approved by the Newcastle Health Authority and University of Newcastle upon Tyne joint ethics committee. All patients and/or their parents gave informed consent before entering the study. The age, sex, body weight, surface area, diagnoses and concomitant chemotherapy for the patients are listed in Table 1. Thirty-five courses of treatment when patients received Pt drugs were studied (18 doses of carboplatin ranging between 340 and 1000 mg $m^{-2}$, 17 doses of cisplatin ranging between 50 and 120 mg $m^{-2}$) in combination with other agents. Cisplatin was administered as a 24-h infusion in normal saline with hydration. Carboplatin was diluted in 100 ml of dextrose solution (5%, w/v) and infused over 60 min. In most cases, the doses of carboplatin and cisplatin administered were based on surface area. Blood samples (3 ml) were obtained from a central line immediately before and at the mid and end points of the infusion, and at 0.25, 0.5, 1, 2, 4, 8 and 24 h after the end of the infusion of carboplatin. For cisplatin, blood samples were collected before and 0.5, 1.0, 2.0, 4.0, 6.0 and 18.0 h into infusion, at the end of infusion and at 0.5, 1.0, 1.5, 2.0 and 24.0 h after the end of infusion. Blood samples were centrifuged at 1000 *g*, 4°C for 10 min. Plasma was then removed and 1 ml was placed in an Amicon Centrifree micropartition unit 30 000 MW cut-off (Amicon, Stonehouse, UK) and centrifuged at 2000 *g* 4°C for 10 min. Plasma ultrafiltrate and plasma were stored at –20°C (< 1 month) until analysed for pharmacokinetic studies. For Pt–DNA adduct analysis, an additional 10 ml of blood was collected into plastic tubes, containing potassium EDTA (20 mg per tube), immediately before treatment with carboplatin or cisplatin and at 6, 24 and 48 h after beginning the infusion of cisplatin or carboplatin. These samples were stored at –80°C (< 1 month) until analysis.

### Pt–DNA adduct level measurement

Blood samples (10 ml) were thawed out gradually on ice over a period of 2–4 h. The blood was transferred to 50-ml tubes and each blood tube was rinsed out with 5–10 ml of sterile distilled water and the washings added to the 50-ml tube. Distilled water was added up to the 25-ml mark on the tube and blood lysis buffer added up to the 50-ml mark. The tube was gently inverted once or twice, placed on ice for 30 min with further occasional mixing, then centrifuged at 2000 *g* for 15 min (4°C) and the supernatant removed down to 5 ml using a pastette. Tubes were refilled with lysis buffer to 50 ml, placed on ice for 10 min with further occasional inversion, and centrifuged as described above. The supernatant was poured off, leaving behind 0.1–0.2 ml of residue. The leucocyte cell pellet was vortex mixed to produce a suspension that was either stored (–20°C) or immediately processed further. The method for the isolation of cellular DNA from frozen pellets of cells was as described previously (Tilby et al, 1991). Pt–DNA adduct levels were determined using a monoclonal antibody (ICR4) specific for cisplatin-induced adducts on DNA (Tilby et al, 1991). The competitive ELISA method used was as described in detail previously (Tilby et al, 1987, 1991).

**Table 1** Characteristics of children studied

| Number | Age (years) | Sex | BW (kg) | SA ($m^2$) | DG | Other drugs | Cisplatin dose (mg $m^{-2}$) | Carboplatin dose (mg $m^{-2}$) |
|---|---|---|---|---|---|---|---|---|
| 1 | 9.3 | M | 19.3 | 0.82 | OS | DOX | 100 | |
| 2 | 9.1 | M | 38.8 | 1.26 | OS | DOX | 100 | |
| 3 | 19.3 | F | 68.8 | 1.75 | OS | DOX | 100 | |
| 4 | 4.6 | F | 19.1 | 0.78 | OS | DOX | 100 | |
| 5 | 17.5 | M | 75.5 | 1.92 | MFHC | DOX | 100 | |
| 6 | 5.4 | F | 18 | 0.78 | MOC | DOX/CPA | 100 | |
| 7 | 13.5 | F | 42.7 | 1.63 | NE | DOX | 120 | |
| 8 | 18.5 | M | 62.7 | 1.8 | OS | DOX | 100 | |
| 9 | 1.2 | M | 8.9 | 0.41 | BSG | VCR | | 580 |
| 10 | 4.7 | M | 17.6 | 0.72 | ME | VCR | | 500 |
| 11 | 11.6 | M | 34.5 | 1.13 | PT | VP16 | | 560 |
| 12 | 3.3 | M | 13.2 | 0.58 | RPR | | | 400 |
| 13 | 1.8 | F | 10.3 | 0.5 | GL | VCR | | 560 |
| 14 | 16.2 | M | 53.3 | 1.57 | PRPD | VP16 | | 600 |
| 15 | 15.4 | M | 43.9 | 1.38 | PT | VP16 | | 500 |
| 15 | | | | | | | | 1000 |
| 16 | 4.1 | F | 13.5 | 0.6 | NB | COJEC | 80 | 750 |
| 17 | 7.5 | M | 22.3 | 0.94 | NB | COJEC | 80 | 700 |
| 18 | *0.5/0.7 | M | *8.4/9.2 | *0.42/0.44 | GL | | 50 | 400 |
| 19 | 3.8 | M | 13.7 | 0.6 | NB | COJEC | 80 | 750 |
| 20 | 5.3 | M | 16.9 | 0.75 | NB | COJEC | 80 | 700 |
| 21 | 1.7 | M | 11.5 | 0.52 | NB | OPEC/OJEC | 80 | 500 |
| 22 | 18.3 | M | 43.2 | 1.4 | NB | COJEC | 80 | 700 |
| 22 | | | | | | | | 780 |
| 23 | 2.4 | M | *12.7/14.3 | *0.57/0.61 | NB | OPEC/OJEC | 100 | 340 |
| 24 | 1.8 | M | *13.5/13.8 | *0.54/0.55 | NB | OPEC/OJEC | 80 | 500 |

© Cancer Research Campaign 1997

**Figure 1** Pt–DNA adduct profiles in PBL of children with cancer after (**A**) a 24-h infusion of cisplatin or (**B**) a 1-h infusion of carboplatin. ▲–▲, ▼–▼, Two children who had adduct measurements after two courses. (**C**): Combined Pt–DNA adduct profiles from the cisplatin and carboplatin groups (mean ± s.d.). **P* < 0.05 compared with 0 h; ***P* < 0.05 compared with carboplatin at 24 h

### Pharmacokinetic studies

Plasma ultrafiltrates prepared after cisplatin or carboplatin treatment were analysed for unbound Pt content by atomic absorption spectrophotometry (AAS) using a graphite tube atomizer (PU9100, ATI Unicam, Cambridge, UK) (Ghazal-Aswad et al, 1996). The typical sensitivity of the AAS was an absorbance of approximately 0.1 units when 20 μl of a standard solution (58 ng Pt $ml^{-1}$, in 0.1 M hydrochloric acid) was analysed, i.e. an aliquot containing about 1 ng of Pt. Each sample was analysed twice and the intra- and interassay coefficients of variation for a quality assurance sample had to be < 10% for an assay to be valid. The samples were diluted with 0.1 M hydrochloric acid to achieve concentrations that were within the range of the standard curve. All drug concentrations described in this paper refer to the intact cisplatin or carboplatin equivalent. The AUC calculated using the trapezoidal rule (Gibaldi and Perrier, 1982) was used for the interpretation of plasma disposition kinetics.

### RESULTS

An attempt was made to measure Pt–DNA adduct levels in every patient receiving either cisplatin or carboplatin as part of the studies described in this paper. Unfortunately, in some cases, inadequate quantities of DNA were obtained for measurement of adduct levels or, alternatively, the patient was too young to allow the collection of sufficient blood. Pt–DNA adduct levels were analysed by competitive ELISA. It has been shown previously that the reaction of DNA with cisplatin in pure solution gives adducts with the same immunoreactivity as those produced when the reaction with DNA has taken place in cells (Tilby et al, 1991). To define the reliable limit of detection of the ELISA for blood samples, a series of DNA samples prepared from blood taken before treatment were tested. The *k*-values ($IC_{50}$) for DNA samples from pretreatment PBLs of children ($n$ = 12) were 3.2 ± 1.0 μg DNA per assay well, corresponding to an apparent level of immunoreactive Pt–DNA adducts of 0.6 ± 0.2 (mean ± sd) nmol Pt $g^{-1}$ DNA. This immunoreactivity was due to interference or the immunological cross-reaction of high concentrations of control DNA in the assay, as reported previously (Tilby et al, 1991). Therefore, it was concluded that 1.0 nmol Pt $g^{-1}$ DNA was the reliable limit for adduct detection, i.e. the mean value for control pretreatment samples plus twice the S.D.

### Pt–DNA adduct levels following cisplatin administration and their relationship to cisplatin dose and pharmacokinetics

Seventeen patients were investigated from whom blood was removed before drug administration and 6, 24 and 48 h after the start of the 24-h infusion of cisplatin. These children received 80–120 mg cisplatin $m^{-2}$ except patient 18 who received 50 mg $m^{-2}$. In all patients, DNA adduct levels were higher at 24 h, ie. at the end of infusion (< 1–5.9 nmol Pt $g^{-1}$ DNA) than at 6 h (< 1–3.2 nmol Pt $g^{-1}$ DNA). Figure 1A shows that for all patients, a similar pattern of adduct levels was observed, with the highest level at 24 h in all patients except patient 23, in whom the highest adduct level was at the 48-h point. Adduct levels declined after the end of the infusion, the average having decreased to about half the level seen at 24 h during the 24 h after the end of the cisplatin infusion.

*© Cancer Research Campaign 1997*

**Figure 2** Relationship between cisplatin dose and increase in apparent Pt–DNA adduct level over the level in the pretreatment sample 6, 24 and 48 h after the start of a 24 h infusion of cisplatin. Points are individual patients

**Figure 3** Relationship between free cisplatin AUC and increase in apparent Pt–DNA adduct level over the level in the pretreatment sample 6, 24 and 48 h after the start of a 24 h-infusion of cisplatin. Points are individual patients and lines are linear regression lines fitted without constraint to the origin. The fitted equations were $y = 13.9x + 0.042$ 6 h and $y = 1.9x + 0.19$ 48 h

The results from the 17 children show considerable interindividual variation. In an attempt to explain this variation, Pt–DNA adduct levels 6, 24 and 48 h after administration of cisplatin were studied for their dependence on either the cisplatin dose or the AUC of unbound cisplatin in plasma. However, there was no clear relationship between adduct levels and cisplatin dose (Figure 2). As shown in Figure 3, there was a weak linear correlation between adduct levels 6 h after the start of cisplatin infusion and the 0–6 h unbound cisplatin AUC ($r^2 = 0.32$, $P < 0.05$), indicating that the unbound cisplatin AUC at early times was a significant determinant of the formation of Pt–DNA adducts. In contrast, there was no linear correlation between adduct levels and the unbound cisplatin AUC at 24 h, i.e. at the end of infusion, and only a very weak relationship at 48 h ($r^2 = 0.22$). Spearman's rank correlation analysis indicated a significant correlation only for the 6- and 48-h time points ($P = 0.009$ and 0.014 respectively).

### Pt–DNA adduct levels after carboplatin administration and their relationship to carboplatin dose and pharmacokinetics

Pt–DNA adduct levels were studied in the DNA of PBLs from 16 children who received carboplatin treatments of 340–1000 mg carboplatin $m^{-2}$ as a 1-h infusion. Figure 1B shows overall data for the apparent Pt–DNA adduct levels for 18 courses (16 patients). The mean immunoreactivity of the DNA increased 2.8-fold between pretreatment and 5 h after the end of infusion. Thereafter, immunoreactivity remained at steady-state until 48 h after the start of the carboplatin infusion. Overall, the Pt–DNA adduct levels after carboplatin were low and frequently at or below the limit of reliable quantification of the ELISA (1 nmol Pt $g^{-1}$ DNA).

The increases in immunoreactivity at 6, 24 and 48 h over pretreatment levels were calculated. Figures 4 and 5 indicate that

© Cancer Research Campaign 1997

**Figure 4** Relationship between carboplatin dose and increase in apparent Pt–DNA adduct level over the level in the pretreatment sample 6, 24 and 48 h after the start of a 1-h infusion of carboplatin. Points are individual patients

**Figure 5** Relationship between free carboplatin AUC and increase in apparent Pt–DNA adduct level over the level in the pretreatment sample 6, 24 and 48 h after the start of a 1-h infusion of carboplatin. Points are individual patients there were no clear relationships between apparent Pt–DNA adduct levels and either carboplatin dose or AUC. This was confirmed by the lack of significance in Spearman's rank correlation analyses, except for the small set of data available for the 48-h time point (Figure 4, $P = 0.03$). This suggests that interpatient differences in immunoreactivity after carboplatin were caused predominantly by factors other than dose or pharmacokinetic variability.

### Comparison of Pt–DNA adduct levels produced by cisplatin and carboplatin

Both carboplatin and cisplatin produced Pt–DNA adducts in a time-dependent manner (Figure 1C). Levels of immunoreactive Pt–DNA adducts appeared to approach a maximum value at the end of the 24-h cisplatin infusion. However, after a 1-h infusion of carboplatin, immunoreactive Pt–DNA adduct levels were lower and did not follow a clear time course. The mean binding of Pt to the DNA of PBLs from children treated with carboplatin at 340–1000 mg m$^{-2}$ was 1.3 nmol Pt g$^{-1}$ DNA, 24 h after the start of the infusion. In contrast, DNA extracted from PBLs of children who had received 50–120 mg m$^{-2}$ cisplatin showed a mean Pt–DNA binding of 3.2 nmol Pt g$^{-1}$ DNA at the end of the 24-h infusion. Thus, although a 20-fold higher unbound drug exposure (AUC) was achieved after carboplatin treatment ($8.0 \pm 3.5$ mg ml$^{-1}$ min vs $0.4 \pm 0.2$ mg ml$^{-1}$ min), the peak level of immunoreactive Pt adducts on the DNA of PBLs from children treated with cisplatin was 2.5-fold greater ($3.2 \pm 1.7$ nmol Pt g$^{-1}$ DNA vs $1.3 \pm 0.6$ nmol Pt g$^{-1}$ DNA).

### Effects of the DNA extraction method

During the course of this work, Ma et al (1995) reported that extraction of DNA from frozen whole-blood samples resulted in higher adduct levels than were observed when white blood cells were

© Cancer Research Campaign 1997

**Figure 6** Model for the proposed reaction of cisplatin and carboplatin with DNA

isolated from the fresh blood sample before freezing. This effect was attributed to the reaction of cisplatin present in the whole blood with DNA in cells that had become permeabilized. The extraction method used in the present work differs in detail to that tested by Ma et al (1995), and certain aspects of the data presented are not consistent with the possibility that the adduct levels were influenced to a large extent by carry-over of active drug in the blood sample. For example, the levels of adducts were lower at 6 h after the start of cisplatin infusion than at 24 h, despite the fact that the concentration of free drug was essentially equal at these two time points. However, to verify that the present results were not influenced by carry-over of active drug, cisplatin and carboplatin were added to samples of freshly obtained blood. The samples were then divided immediately and either frozen and extracted by our standard method, or processed according to the buffy coat method of Ma et al (1995). DNA extracted from frozen whole blood containing 1 μM cisplatin (representing the mean concentration of free drug present in blood samples removed for adduct measurements) was not significantly more immunoreactive than DNA from blood not treated with platinum complexes. DNA extracted from frozen whole blood containing 5 μM cisplatin (twofold higher than was present in any blood sample taken for adduct measurements) showed a slight increase in immunoreactivity but this only just attained the level of significance of 1 nmol Pt $g^{-1}$ DNA. No blood sample taken for the measurement of DNA adducts induced by carboplatin was taken until at least 5 h after the end of carboplatin infusion and therefore, unlike the situation with cisplatin, the level of reactive drug had declined markedly before removal of the first sample for measurement of adducts. DNA extracted from whole-blood to which carboplatin had been added to give concentrations up to 50 μM (five times the maximum free drug concentration present in blood samples removed for adduct measurements) was not significantly more immunoreactive than DNA from blood not treated with platinum complexes. None of the blood samples processed by the buffy coat method of Ma et al showed detectable increases in immunoreactivity above the control DNA.

### DISCUSSION

The objectives of this study were to describe Pt–DNA adduct formation in PBLs of children with cancer, and to investigate the relationship between adduct levels and both the doses and pharmacokinetics of cisplatin and carboplatin. Reed et al (1990) have reported that in a group of relapsed ovarian cancer patients the extent of Pt–DNA adduct formation in white blood cells (WBC) DNA was directly related to disease response after treatment with either single-agent cisplatin or carboplatin. Furthermore, adduct levels in leucocytes were more closely related to disease response than other previously identified prognostic variables, including patient performance status, stage of disease, response to previous treatment, total previous Pt drug dose, age, histological type and grade. Analogous observations were reported for patients with testicular cancer by the same group (Reed et al, 1988) and by Fichtinger-Schepman et al (1990). More recently, in patients with a variety of tumour types, Blommaert et al (1993) found that immunohistological staining of buccal cells for carboplatin-induced adducts was significantly higher in partial responders than in non-responders. Measurement of PBL Pt–DNA adduct levels by AAS also demonstrated higher levels in responding vs non-responding patients (Parker et al, 1991) and it has been reported that response to single agent cisplatin, or cisplatin plus etoposide, was related to PBL adduct levels measured by AAS in a mixed group of 43 patients (Ma et al, 1994; Schellens et al, 1996). These separate studies support the concept that there may be a 'parallel' between malignant tumour tissues and normal cells, including PBLs, in their capacity to form, repair, or otherwise retain Pt–DNA adducts. However, as most of the above studies did not include pharmacokinetic measurements, the possibility cannot be excluded that the variation in adduct levels in PBLs was simply a reflection of pharmacokinetic variability. Indeed, in support of this latter suggestion, the study described by Ma et al (1994) and Schellens et al (1996) did show a relationship between both adduct levels and cisplatin AUC, and AUC and response, as well as adduct levels and response.

There were substantial differences in absolute adduct levels between the above mentioned clinical studies. In addition to the drug used, the dose administered and the pharmacokinetics, these differences may be related to dosing schedules, assay methodology and/or other factors. The immunoassay used here, readily detected adducts at the levels found in PBLs of paediatric patients receiving conventional doses of cisplatin. Pt–DNA adduct levels ranged up to 5.9 nmol $g^{-1}$ DNA, values much higher than those determined by Reed (1990) (up to 0.4 nmol $g^{-1}$ DNA), but similar to those determined by Fichtinger-Schepman (1990) (up to 10 nmol $g^{-1}$ DNA) and Ma (1995) (mean values 13 and 7 nmol $g^{-1}$ DNA). In the case of carboplatin, adduct levels were generally low and were only just detectable by the ELISA. The low levels of adducts found in the patients after carboplatin treatment could be due to either lower levels of Pt–DNA binding or qualitative differences in the adducts formed by cisplatin and carboplatin. These possibilities are discussed more fully below.

Levels of cisplatin adducts increased during the 24-h infusion and then declined over the next 24 h (Figures 1A and C). Observed levels of carboplatin-induced adducts often increased between 6 and 24 h (Figure 1B), despite the fact that the drug was only infused over 1 h and that unbound drug was largely cleared from the circulation by 6 h. Furthermore, between 24 h and 48 h after infusion the observed levels of adducts showed no appreciable reduction. This difference in time course of adduct level change might be explained by the post-infusion conversion of monofunctional carboplatin-DNA products into bidentate adducts (Figure 6). Because of the much longer infusion time for cisplatin compared with carboplatin, most of the initially formed cisplatin adducts had a longer time period in which to complete any delayed second-arm reactions before removal of the first, post-infusion blood sample.

© Cancer Research Campaign 1997

However, the main cause of the observed difference between the drugs is likely to be the slower rate of the second-arm reaction for carboplatin compared with cisplatin (Knox et al, 1986). Slow loss of the cyclobutanedicarboxylato group from monofunctional adducts on DNA and guanosine monophosphate was shown by Knox et al (1986) and Frey et al (1993) respectively. The immunoreactivity to ICR4 of the ultimate reaction products of carboplatin with DNA was the same as the immunoreactivity of cisplatin adducts (Tilby et al, 1991; B Peng et al, unpublished data), although recent data indicate slight differences in the distribution of adduct types formed by the two drugs (Fichtinger-Schepman et al, 1995). The monoclonal antibody ICR4 was raised against cisplatin-DNA adducts and probably does not recognize the monovalent carboplatin–DNA adduct (Ghazal-Aswad et al, 1993).

In the present study, after the administration of 'clinically equivalent' doses of cisplatin and carboplatin, carboplatin was found to produce lower levels of immunoreactive Pt–DNA adduct than cisplatin in PBLs. The average peak level of immunoreactive adducts formed during cisplatin therapy was 2.5-fold higher than that observed after carboplatin therapy despite the carboplatin plasma AUC being 20-fold higher than the cisplatin AUC. Cisplatin adduct levels decreased markedly during the 24 h after the end of the drug infusion (Figure 1C), possibly due to DNA repair processes. The longer infusion of cisplatin (24 h) compared with carboplatin (1 h) provided greater opportunity for initially formed adducts to be removed before the first post-infusion blood sample was taken. This factor may have acted to reduce the detectable levels of adducts formed by cisplatin and thereby diminish the detected differences between the two drugs.

During the course of this work, Ma et al (1995) reported that extraction of DNA from whole blood, as opposed to isolated white blood cells, could result in elevated adduct levels that were attributed to the effects of carried over active drug present in the blood sample. The data presented here were not significantly influenced by this phenomenon, although cisplatin at concentrations higher than were present in any of the blood samples taken for adduct measurement did show a slight effect. Compared with the samples studied by Ma et al, the concentrations of cisplatin in the present samples were probably lower because of the use of a 24 h-infusion. This, together with slight differences in the extraction technique apparently avoided the potential discrepancy reported by Ma et al, however, our results indicated that this problem would become significant at higher plasma concentrations of cisplatin and are thereby consistent with the findings of Ma et al (1995).

Previous studies showing interpatient variation in Pt–DNA adduct levels were summarized in the introduction. In the present study, measurements have been made of both Pt–DNA adducts and of unbound plasma drug levels. The results show that variation in pharmacokinetics could account for only a small proportion of the interpatient variation in adduct levels. Indeed, the only convincing evidence for a relationship between drug plasma AUC and adduct level was seen in patients who received cisplatin treatment, where a weak correlation between Pt–DNA adduct levels at 6 h and the 0–6 h cisplatin AUC was observed (Figure 3). Thus, cisplatin AUC may be a determinant of Pt–DNA adduct levels at early time points. At later time points (24 and 48 h), no clear relationship between cisplatin AUC and adduct levels was seen in the present study. The lack of a relationship at later time points could reflect the impact of either removal of adducts (DNA repair) and/or the turnover of PBLs on total blood Pt–DNA adduct levels. In the case of carboplatin, there was no relationship between adduct level and carboplatin AUC at any of the time points studied, which is in agreement with the observation by Blommaert et al (1993), in which in a combination study the increase in drug-induced nuclear staining was not related to the dose of either carboplatin or cisplatin. Together these data suggest that adduct levels after carboplatin are determined primarily by factors other than drug exposure, which could include drug uptake, reaction with glutathione or other inactivation mechanisms and DNA repair (de Graeff et al, 1988).

As discussed above, Pt–DNA adduct levels in PBLs and tumour response have been shown to be related in a number of studies (Reed et al, 1986, 1987, 1990; Fichtinger-Schepman et al, 1990; Ma et al, 1994). If low adduct levels in poor responders are due to an intracellular factor in the PBLs then the same factor must be operating in the tumour cells, i.e. the tumour cell is exhibiting a host phenotype. The numbers of patients studied here were too small to permit the relationship between adduct levels and response to be studied, particularly as the Pt drugs formed only part of complex chemotherapy protocols and the disease types and their chemosensitivity varied widely (Table 1).

In conclusion, this study demonstrates that the variation in Pt–DNA adduct formation seen in peripheral blood cells of children treated with cisplatin and carboplatin is not related purely to dose administered or the unbound drug AUC. There was a marked difference in the levels and kinetics of DNA modification produced by cisplatin and carboplatin treatment such that, after standard clinical doses, cisplatin produced higher levels of immunoreactive Pt–DNA adduct than carboplatin despite the doses and AUCs for carboplatin being higher than for cisplatin. All of the children studied in this paper were receiving cisplatin and/or carboplatin in combination with other cytotoxic agents and it was not possible to study the relationship between Pt–DNA adduct levels and response or toxicity. To define the relevance of the Pt–DNA adduct levels determined in patients, further in vitro and clinical studies of the relationship between toxicity, activity and Pt–DNA adduct formation are warranted.

### ABBREVIATIONS

BW, body weight; SA, surface area; DG, diagnosis; F, female; M, male; OS, osteosarcoma; MFHC, malignant fibrous histiocytosis; MOC, mucoepidermoid carcinoma of the parotid gland; NE, neuroendocrine tumour; BSG, brainstem glioma; ME, medulloblastoma; PT, pineal teratoma; RPR, relapsed parameningeal rhabdomyosarcoma; GL, glioma; PRPD, peritoneal relapse of pineal dysgerminoma; NB, neuroblastoma; DOX, doxorubicin; CPA, cyclophosphamide; VCR, vincristine; VP16, etoposide; COJEC, treatment with carboplatin, vincristine and VP16 on day 1, and with cisplatin and vincristine on day 10; OPEC/OJEC, treatment with cisplatin, vincristine, VP16 and cyclophosphamide on day 1 and with carboplatin, vincristine, VP16 and cyclophosphamide on day 21. *Two values, the measured age, BW and SA at the time when cisplatin and carboplatin studies were performed respectively.

### ACKNOWLEDGEMENTS

We thank the North of England Cancer Research Campaign, Leukaemia Research Fund and the North of England Children's Cancer Research Fund for supporting this study, and Johnson-Matthey for providing us with cisplatin and carboplatin. We would also like to thank the patients and parents who took part in this study, and the nursing and medical staff at the Royal Victoria Infirmary and Newcastle General Hospital who cared for them.

© Cancer Research Campaign 1997

### REFERENCES

Blommaert FA, Michael C, Terheggen PMAB, Muggia FM, Kortes V, Schornagel JH, Hart AAM and Den Engelse L (1993) Drug-induced DNA modification in buccal cells of cancer patients receiving carboplatin and cisplatin combination chemotherapy, as determined by an immunocytochemical method: Interindividual variation and correlation with disease response. *Cancer Res* **53**: 5669–5675

Campbell AB, Kalman SM and Jacobs C (1983) Plasma platinum levels: Relationship to cisplatin dose and nephrotoxicity. *Cancer Treat Rep* **67**: 169–172

Chatelut E, Boddy AV, Peng B, Rubie H, Lavit M, Dezeuze A, Pearson ADJ, Roche H, Robert A, Newell DR and Canal P (1996) Population pharmacokinetics of carboplatin in children. *Clin Pharmacol Ther* **59**: 436–443

De Graeff A, Slebos RJC and Rodenhuis S (1988) Resistance to cisplatin and analogues: Mechanisms and potential clinical implications. *Cancer Chemother Pharmacol* **22**: 325–332

Doz F and Pinkerton R (1994) What is the place of carboplatin in paediatric oncology? *Eur J Cancer* **30A**: 194–201

Egorin MJ, Van Echo DA, Tipping SJ, Olman EA, Whitacre MY, Thompson BW and Aisner J (1984) Pharmacokinetics and dosage reduction of cis-diammine(1,1-cyclobutanedicarboxylato)platinum in patients with impaired renal function. *Cancer Res* **44**: 5432–5438

Fichtinger-Schepman AMJ, Baan RA, Luiten-Schuite A, Van Dijk M and Lohman PHM (1985) Immunochemical quantitation of adducts induced in DNA by cis-diammine-dichloroplatinum(II) and analysis of adduct-related DNA-unwinding. *Chem Biol Interactions* **55**: 275–288

Fichtinger-Schepman AMJ, Van der Velde-Visser SD, Van Dijk-Knijnenburg HCM, Van Oosterom AT, Baan RA and Berends F (1990) Kinetics of the formation and removal of cisplatin-DNA adducts in blood cells and tumour tissue of cancer patients receiving chemotherapy: Comparison with in vitro adduct formation. *Cancer Res* **50**: 7887–7894

Fichtinger-Schepman AMJ, Van Dijkknijnenburg HCM, Van der Velde-Visser SD, Berends F and Baan RA (1995) Cisplatin- and carboplatin-DNA adducts: Is Pt-AG the cytotoxic lesion? *Carcinogenesis* **16**: 2447–2453

Frey U, Ranford JD and Sadler PJ (1993) Ring-opening reactions of the anticancer drug carboplatin: NMR characterization of cis-[Pt($NH_3$)$^2$ (CBDCA-O)(5′-GMP-N7)] in solution. *Inorg Chem* **32**: 1333–1340

Ghazal-Aswad S, Tilby MJ, Newell DR, Knox R, Errington W, Sinha DP and Calvert AH (1993) Comparison of DNA adducts formed by cisplatin and carboplatin in vitro and in vivo by immunoassay. *Br J Cancer* **67**(suppl. XX): 81

Ghazal-Aswad S, Calvert AH and Newell DR (1996) A single-sample assay for the estimation of the area under the free carboplatin plasma concentration versus time curve. *Cancer Chemother Pharmacol* **37**: 429–434

Gibaldi M and Perrier D (1982) *Pharmacokinetics*, 2nd edn, pp. 409–417. Marcel Dekker: New York

Harland SJ, Gumbrell LA and Horwich A (1991) Carboplatin dose in combination chemotherapy for testicular cancer. *Eur J Cancer* **27**: 691–695

Horwich A, Dearnaley DP, Nicholls J, Jay G, Mason M, Harland S, Peckham MJ and Hendry WF (1991) Effectiveness of carboplatin, etoposide, and bleomycin combination chemotherapy in good-prognosis metastatic testicular nonseminomatous germ cell tumors. *J Clin Oncol* **9**: 62–69

Jodrell DI, Egorin MJ, Canetta RM, Langenberg P, Goldbloom EP, Burroughs JN, Goodlow JL, Tan S and Wiltshaw E (1992) Relationships between carboplatin exposure and tumor response and toxicity in patients with ovarian cancer. *J Clin Oncol* **10**: 520–528

Knox RJ, Friedlos F, Lydall DA and Roberts JJ (1986) Mechanism of cytotoxicity of anticancer platinum drugs: Evidence that cis-diamminedichloroplatinum(II) and cis-diammine-(1,1-cyclobutanedicarboxylato) platinum(II) differ only in the kinetics of their interaction with DNA. *Cancer Res* **46**: 1972–1979

Ma J, Verweij J, Planting ASTH, De Boer-Dennert M, Van der Burg MEL, Stoter G and Schellens JHM (1994). Pharmacokinetic-dynamic relationship of weekly high dose cisplatin (C) in solid tumor patients (PTS). *Proc ASCO* **13**: 133

Ma J, Verweij J, Planting ASTH, De Boer-Dennert M, Van Ingen HE, Van der Burg MEL, Stoter G and Schellens JHM (1995) Current sample handling methods for measurement of platinum-DNA adducts in leucocytes in man lead to discrepant results in DNA adduct levels and DNA repair. *Br J Cancer* **71**: 512–517

Marina NM, Rodman J, Shema SJ, Bowman LC, Douglass E, Furman W, Santana VM, Hudson M, Wilimas J, Meyer W, Madden T and Pratt C (1993) Phase I study of escalating targeted doses of carboplatin combined with ifosfamide and etoposide in children with relapsed solid tumors. *J Clin Oncol* **11**: 554–560

Newell DR, Siddik ZH, Gumbrell LA, Boxall FE, Gore ME, Smith IE and Calvert AH (1987) Plasma free platinum pharmacokinetics in patients treated with high dose carboplatin. *Eur J Cancer Clin Oncol* **23**: 1399–1405

Newell DR, Pearson ADJ, Balmanno K, Price L, Wyllie RA, Keir M, Calvert AH, Lewis IJ, Pinkerton CR and Stevens MC (1993) Carboplatin pharmacokinetics in children: The development of a pediatric dosing formula. *J Clin Oncol* **11**: 2314–2323

Parker RJ, Gill I, Tarone R, Vionnet JA, Grunberg S, Muggia FM and Reed E (1991) Platinum-DNA damage in leukocyte DNA of patients receiving carboplatin and cisplatin chemotherapy, measured by atomic absorption spectrometry. *Carcinogenesis* **12**: 1253–1258

Pearson AD, Craft AW, Pinkerton CR, Meller ST and Reid MM (1992) High-dose rapid schedule chemotherapy for disseminated neuroblastoma. *Eur J Cancer* **28A**: 1654–1659

Pinkerton CR, Pritchard J and Spitz L (1986) High complete response rate in children with advanced germ cell tumors using cisplatin-containing combination chemotherapy. *J Clin Oncol* **4**: 194

Poirier MC, Lippard SJ, Zwelling LA, Ushay HM, Kerrigan D, Thill CC, Santella RM, Grunberger D and Yuspa SH (1982) Antibodies elicited against cis-diamminedichloroplatinum(II)-modified DNA are specific for cis-diamminedichloro-platinum(II)-DNA adducts formed in vivo and in vitro. *Proc Natl Acad Sci USA* **79**: 6443–6447

Reece PA, Stafford I, Russell J, Khan M and Gill PG (1987) Creatinine clearance as a predictor of ultrafilterable platinum disposition in cancer patients treated with cisplatin: Relationship between peak ultrafilterable platinum plasma levels and nephrotoxicity. *J Clin Oncol* **5**: 304–309

Reed E, Yuspa SH, Zwelling LA, Ozols RF and Poirier MC (1986) Quantitation of cis-diamminedichloroplatinum (II) (Cisplatin)-DNA-intrastrand adducts in testicular and ovarian cancer patients receiving cisplatin chemotherapy. *J Clin Invest* **77**: 545–550

Reed E, Ozols RF, Tarone R, Yuspa SH and Poirier MC (1987) Platinum-DNA adducts in leukocyte DNA correlate with disease response in ovarian cancer patients receiving platinum-based chemotherapy. *Proc Natl Acad Sci USA* **84**: 5024–5028

Reed E, Ozols RF, Tarone R, Yuspa SH and Poirier MC (1988) The measurement of cisplatin-DNA levels in testicular cancer patients. *Carcinogenesis* **9**: 1909–1911

Reed E, Ostchega Y, Steinberg SM, Yuspa SH, Young RC, Ozols RF and Poirier MC (1990) Evaluation of platinum-DNA adduct levels relative to known prognostic variables in a cohort of ovarian cancer patients. *Cancer Res* **50**: 2256–2260

Roberts JJ and Thomson AJ (1979) The mechanism of action of antitumour platinum compounds. *Prog Nucleic Acid Res Mol Biol* **22**: 71–133

Schellens JHM, Ma J, Planting ASTh, van der Burg MEL, van Meerten E, de Boer-Dennert M, Schmitz PIM, Stoter G and Verweij J (1996) Relationship between the exposure to cisplatin, DNA-adduct formation in leucocytes and tumour response in patients with solid tumours. *Br J Cancer* **73**: 1569–1575

Sherman SE and Lippard SJ (1987) Structural aspects of platinum anticancer drug interactions with DNA. *Chem Rev* **87**: 1153–1181

Sorensen BT, Stromgren A, Jakobsen P and Jakobsen A (1991) Dose-toxicity relationship of carboplatin in combination with cyclophosphamide in ovarian cancer patients. *Cancer Chemother Pharmacol* **28**: 397–401

Terheggen PMAB, Dijkman R, Begg AC, Dubbelman R, Floot BGJ, Hart AAM and Den Engelse L (1988) Monitoring of interaction products of cis-diamminedichloroplatinum(II) and cis-diammine(1, 1-cyclobutane-dicarboxylato)platinum(II) with DNA in cells from platinum-treated cancer patients. *Cancer Res* **48**: 5597–5603

Thomas DJ, Clifford SC, Aherne W, Neal DE and Newell DR (1994) Pharmacokinetic determinants of response in bladder cancer. *Br J Cancer* **69**(suppl. XXI): 30

Tilby MJ, Styles JM and Dean CJ (1987) Immunological detection of DNA damage caused by melphalan using monoclonal antibodies. *Cancer Res* **47**: 1542–1546

Tilby MJ, Johnson C, Knox RJ, Cordell J, Roberts JJ and Dean CJ (1991) Sensitive detection of DNA modifications induced by cisplatin and carboplatin in vitro and in vivo using a monoclonal antibody. *Cancer Res* **51**: 123–129

© Cancer Research Campaign 1997